# The complex existence of γδ T cells following transplantation: the good, the bad and the simply confusing

**DOI:** 10.1002/cti2.1078

**Published:** 2019-09-17

**Authors:** Lucy C Sullivan, Evangeline M Shaw, Sanda Stankovic, Gregory I Snell, Andrew G Brooks, Glen P Westall

**Affiliations:** ^1^ Department of Microbiology and Immunology The University of Melbourne at The Peter Doherty Institute for Infection and Immunity Melbourne VIC Australia; ^2^ Lung Transplant Service The Alfred Hospital Melbourne VIC Australia

**Keywords:** gamma delta T cells, transplant immunology, graft‐versus‐host disease

## Abstract

Gamma delta (γδ) T cells are a highly heterogeneous population of lymphocytes that exhibit innate and adaptive immune properties. Despite comprising the majority of residing lymphocytes in many organs, the role of γδ T cells in transplantation outcomes is under‐researched. γδ T cells can recognise a diverse array of ligands and exert disparate effector functions. As such, they may potentially contribute to both allograft acceptance and rejection, as well as impacting on infection and post‐transplant malignancy. Here, we review the current literature on the role and function of γδ T cells following solid organ and hematopoietic stem cell transplantation.

## Introduction

Gamma delta (γδ) T cells consist of ~4% of the total T cell population in human peripheral blood; however, they typically comprise a higher proportion of T cells in skin and mucosal epithelium.^1^ γδ T cells are a highly heterogenous group of lymphocytes that display broad functional abilities, interacting with both innate and adaptive immune compartments. A body of evidence indicates that γδ T cells are important in tissue homeostasis and repair, both in the skin and mucosa.^2^ Mice deficient in γδ T cells spontaneously develop inflammatory bowel disease^3^ and succumb to dextran sodium sulphate‐induced colitis (reviewed by Nanno *et al*.^4^). In addition, through the production of TGFβ, γδ T cells limit damage to renal epithelial cells in a rat model of autoimmune‐mediated glomerulonephritis^5^ and protect pulmonary epithelial cells from damage after ozone exposure.^6^ Furthermore, studies on antigenic tolerance in animal models have shown dependence on γδ T cells.^7^

Gamma delta T cells are mediators of both anti‐inflammatory and pro‐inflammatory responses. γδ T cells exert their effects largely through MHC‐independent mechanisms and can be directly cytotoxic but can also be activated by other immune cells. Furthermore, γδ T cells appear to be central in the control of post‐transplant infection, particularly to cytomegalovirus (CMV). Their role in transplantation outcome remains unclear, with evidence suggesting they can be both effectors and suppressors of allogenic rejection, but nonetheless highlighting them as an important component of the post‐transplant immune response.

## γδ T cell receptors, ligands and effector functions

Gamma delta T cells are controlled by a suite of cell‐surface expressed molecules, including a T cell receptor (TCR) and several receptors more commonly associated with natural killer (NK) cells. The loci encoding the γδ TCR genes are the T cell receptor gamma (TRG, encoding the gamma chain) and T cell receptor delta (TRD, encoding the delta chain).^8^ Largely analogous to classical αβ T cells, TCR rearrangement is dependent on the expression of recombination activating genes (RAG). However, compared to αβ T cells, the repertoire of γδ V and J gene segments is restricted, with the TRG locus containing only 12 Variable (V) segment genes, of which 6 are functional, and the TRD locus containing eight functional V region genes. This is in comparison with αβ T cells, which have 52 Vβ genes and 70 Vα genes.^9^ Furthermore, of the TRD genes, only four of these are frequently used: Vδ1, Vδ2, Vδ3 and Vδ5. However, γδ TCR still has extreme sequence variation because of a high degree of junctional diversity as a result of D segment rearrangement.^10^ Also, unlike αβ T cells, the vast majority of γδ T cells do not express either the CD4 or CD8 co‐receptor. Important in the context of transplantation, γδ T cells with different TCR localise to distinct regions. The vast majority of healthy adult peripheral blood γδ T cells are Vγ9Vδ2, whereas γδ T cells bearing Vδ1, Vδ3 or Vδ5 TCR are located in the skin, intestine, lung and liver.^11^, ^12^

In addition to their TCR, γδ T cells express many receptors in common with NK cells. The NK cell receptor NKG2D is expressed on a large proportion of γδ T cells and recognises the stress‐inducible ligands MHC class I chain‐related proteins (MIC)‐A and (MIC)‐B and UL16 binding proteins (ULBPs), many of which may be upregulated following transplantation.^13^, ^14^, ^15^ Vδ1 cells reportedly recognise MIC‐A via both TCR and NKG2D, although TCR interactions were not involved in their cytotoxic activity^16^ (Table 1). Cytotoxic activity is also triggered by Vγ9Vδ2 γδ T cells upon ligation of another NK cell receptor, DNAM‐1^17^ (Table 1). Subsets of γδ T cells also express other NK cell receptors, including NKp30, NKp44 and CD94‐NKG2 receptors.^18^ Another important receptor also shared with NK cells is CD16, a low‐affinity receptor for the constant region of IgG. The expression of CD16 allows γδ T cells to recognise IgG opsonised pathogens or target cells without a strict requirement for TCR engagement.^19^

**Table 1:** Human γδ T cell ligands and co‐expressed receptors

γδ TCR subset	Anatomical location	TCR ligand	Co‐expressed receptors	References
Vγ9Vδ2	PB	Phosphoantigens	NKG2D, DNAM‐1	17, 20
Vδ1	PB, skin, gut, spleen, liver	CD1 family, MIC‐A/B, ULBPs	NKG2D, NKp30, CD16	16, 19, 22, 23
Vδ3	PB, liver	CD1d		24
Vγ8Vδ3	PB	Annexin A2		25
Vγ4Vδ5	PB	Endothelial protein C receptor		21

MIC, MHC class I chain‐related protein; PB, peripheral blood; TCR, T cell receptor; ULBPs, UL16 binding proteins.

In contrast to αβ T cells, γδ T cells typically do not recognise ligands in the context of MHC molecules. Of the known ligands, Vγ9Vδ2 γδ T cells are activated by phosphoantigens, which can be produced by microbes or as a result of malignant transformation,^20^ whereas Vγ4Vδ5 TCRs bind to endothelial protein C receptor (EPCR)^21^ (Table 1). The ligands for Vδ1 cells have remained somewhat more elusive, but are reported to include MHC‐like molecules, such as the CD1 family^22^ and MIC‐A/B^23^ (Table 1). Another member of the CD1 family, CD1d, is recognised by subsets of Vδ3 γδ T cells,^24^ whereas other subsets of Vδ3 cells recognise annexin A2^25^ (Table 1). The ligands for TCR of other γδ T cells are still largely undefined.

Interestingly, γδ T cell effector function depends on their niche. For example, intestinal epithelium‐resident γδ T cells produce keratinocyte growth factor, contributing to the intestinal barrier health and homeostasis.^26^ Firmly placed at the interface of innate and adaptive immunity, following recognition of ligands by the TCR and/or activating NK cell receptors, γδ T cells are potent producers of pro‐inflammatory cytokines (IFN‐γ, TNF‐α, IL‐17) and can directly lyse infected or transformed cells via perforin‐ and granzyme‐dependent mechanisms. Following activation, γδ T cells can also induce several cell types into antigen‐presenting cells, thereby promoting dendritic cell maturation, CD4^+^ and CD8^+^ T cell priming, as well as antibody production.^27^ γδ T cells can also produce inflammatory and chemotactic chemokines such as RANTES, CXCL10 and lymphotactin. They are also capable of cross‐presenting antigens, thereby inducing CD8^+^ T cell responses.^28^ In addition, γδ T cells do not require TCR engagement for cytokine production. Instead, they can be activated to produce IL‐17 by cytokines such as IL‐1β and IL‐23.^29^

In summary, given the complexity of receptors expressed, ligands bound and responses exerted by γδ T cells, it is not surprising that they have been implicated as playing diverse roles in transplantation outcome.

## Evidence for γδ T cells in adverse outcomes following transplantation

**Figure 1:**
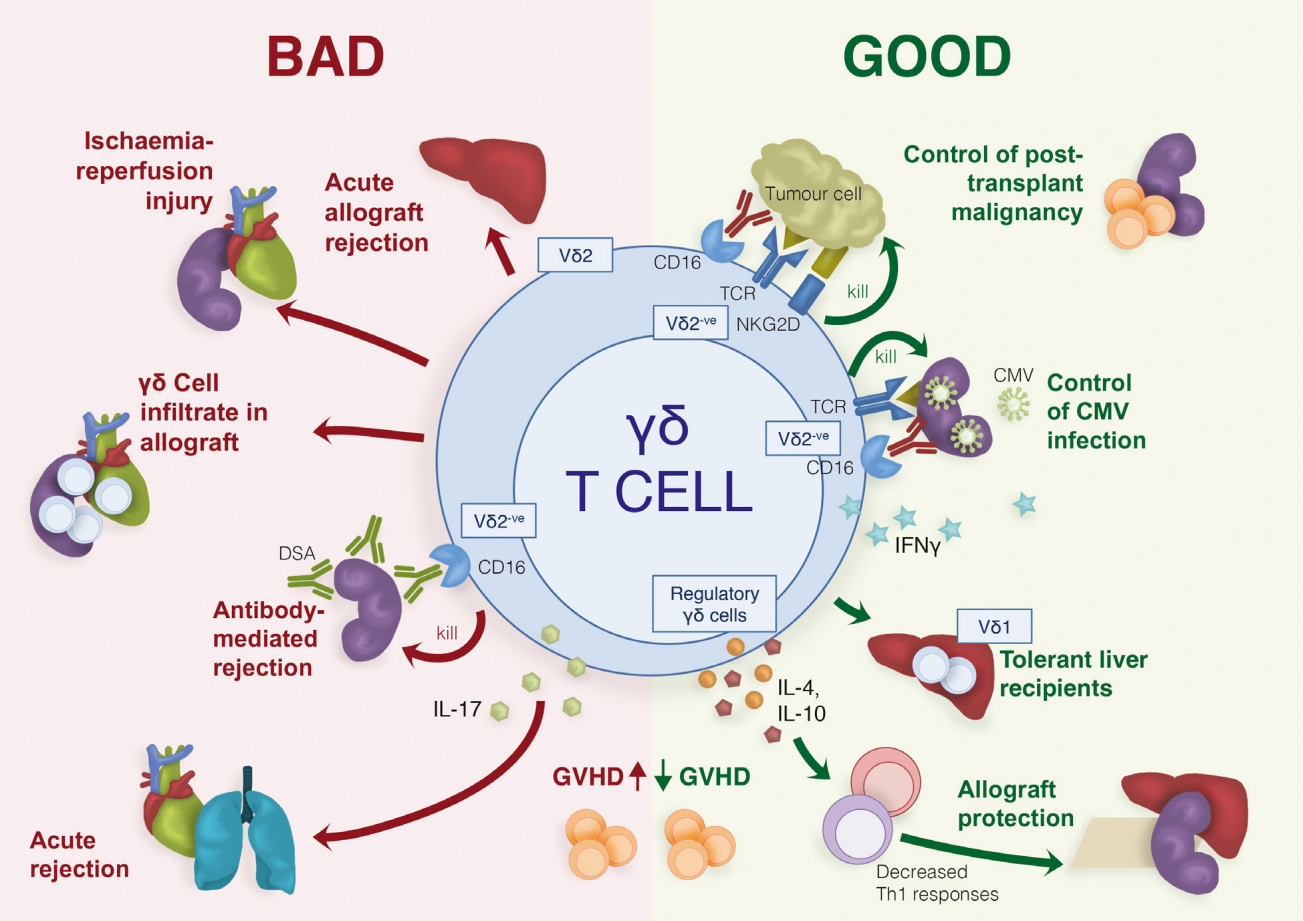
Gamma delta T cells in transplantation: the good, the bad and the simply confusing. Adverse and favorable associations between γδ T cells and outcomes following transplantation have been reported. Left: ‘Bad’ outcomes in animal studies include ischaemia‐reperfusion injury (in heart and kidney), and acute rejection of heart and lung allografts via IL‐17‐mediated mechanisms. Other adverse outcomes in humans include the presence of γδ T cell infiltration in kidney and heart allografts; the presence of Vδ2 γδ T cells in patients with liver allograft rejection; antibody‐mediated rejection mediated by Vδ2‐negative (Vδ2^−^) γδ T cells via recognition of donor‐specific antibodies (DSA) through CD16, and an *increased* incidence of graft‐versus‐host disease (GvHD). Right: ‘Good’ outcomes include *decreased* GvHD incidence; increase in Vδ1‐positive (Vδ1^+^) infiltration in tolerant liver recipients; secretion of IL‐4 and IL‐10 leading to allograft protection (observed in skin, kidney and liver); control of cytomegalovirus (CMV) infection by Vδ2^−^ cells via IFNγ and the killing of infected cells through their T cell receptor (TCR) or CD16 engagement; and control of post‐transplant malignancies by Vδ2^−^ cells which recognise tumor cells through CD16, TCR or other receptor engagements.

A large proportion of the research implicating γδ T cells in adverse outcomes following transplantation comes from small animal models (Figure 1). Although γδ T cell phenotypes and function in mice and humans are broadly consistent, there are also distinct differences between species, most notably the types of TCR ligands that have been identified (Table 2).

**Table 2:** A comparison of mouse and human γδ T cells

	Mouse	Human	References
Tissue distribution	0.5–10% of lymphocytes in secondary lymphoid organs and blood; higher in intestinal epithelium, skin, reproductive organs	0.5–10% of lymphocytes in secondary lymphoid organs and blood, lung, skin, liver; higher in intestinal epithelium	75, 76, 77, 78
Predominant TCR V gene segment by location	Vγ1, Vγ4 in blood; Vδ1 in skin, mucosa of the female reproductive tract, lung; Vδ4 in intestinal epithelium	Vδ2 in blood; Vδ1 in peripheral tissues	12, 79, 80
Effector functions	Direct cytotoxicity; can produce a broad spectrum of cytokines associated with Th1, Th2, Th17 and T reg	Direct cytotoxicity/ADCC; can produce a broad spectrum of cytokines associated with Th1, Th2, Th17 and T reg	19, 65, 81, 82, 83, 84, 85
TCR ligands	CD1d‐restricted phospholipids; Qa1‐restricted peptides; direct recognition of viral proteins (e.g. HSV‐1 glycoprotein I)	Vδ2‐phosphoantigens (butyrophilin‐dependent); Vδ1‐stress‐induced self‐antigens (e.g. MIC‐A/B); glycolipids presented by CD1c/d	86, 87, 88, 89, 90, 91, 92, 93
NKG2D ligands	Rae‐1, H60, MULT1	MIC‐A/B, ULBP1‐6	94, 95, 96, 97, 98
NKG2A ligands	Qa‐1 (inhibitory)	HLA‐E (inhibitory)	99, 100, 101
Role in anti‐CMV response	γδ T cells provide protection from CMV‐induced death	Vδ2^−^ γδ T cells show effector functions against CMV‐infected cells	48, 56, 102

ADCC, antibody‐dependent cell‐mediated cytotoxicity; CMV, cytomegalovirus; MIC, MHC class I chain‐related protein; TCR, T cell receptor.

In small animal models, γδ T cells have been implicated in playing a role in ischaemia‐reperfusion injury (IRI). This has been demonstrated by reduced IRI in TCR γδ‐deficient mice in a model of kidney transplantation^30^ and the observation that IL‐17A, produced by γδ T cells, is elevated in a mouse model of cardiac transplantation.^31^ However, the proposed mechanisms differ between the studies, with γδ T cells either inducing the recruitment αβ T cells into the allograft,^30^, ^32^ or alternatively by inducing neutrophil recruitment through the production of IL‐17.^31^ The production of IL‐17 from γδ T cells also is reported to contribute to acute and chronic allograft dysfunction in small animal models of skin,^33^ heart^34^, ^35^, ^36^ and lung^37^ transplantation. However, in the mouse model of lung transplantation, despite being potent producers of intragraft IL‐17, there was no effect of γδ T cell depletion on the development of acute rejection or fibrosis.^37^ In addition, the literature is void of a link between IL‐17 producing γδ T cells and rejection following solid organ transplantation in humans.

There is also a disconnect between animal studies and human transplantation with respect to the role of γδ T cells in graft‐versus‐host disease (GvHD) following hematopoietic stem cell transplantation (HSCT). Early animal studies linked γδ T cells to the progression of GvHD. For example, Blazar and others^38^ created a transgenic mouse model where a large proportion of T cells expressed the γδ TCR. These transgenic cells proliferated and killed mismatched cells *in vitro*. Moreover, when the transgenic cells were infused into mismatched mice following bone‐marrow transplantation, they infiltrated GvHD target tissues, indicating their capacity to cause pathology.^38^ Another early study in mice revealed that depletion of γδ T cells resulted in reduced GvHD.^39^ However, the evidence for γδ T cells contributing to GvHD following HSCT in humans is varied. While some studies showed that higher numbers of γδ T cells were correlated with increased incidence of acute GvHD,^40^, ^41^ other studies have either found no correlation between numbers of γδ T cells and GvHD^42^ or that lower numbers were associated with increased incidence of GvHD.^43^ However, it is also possible that only specific subsets of γδ T cells adversely contribute to GvHD, notably Vδ2 γδ T cells which were implicated in the study by Viale *et al*.^40^

Interestingly, these same Vδ2 γδ T cells may also be associated with poorer outcomes following solid organ transplantation. Yu *et al*.^44^ showed higher proportions of Vδ2 cells in liver transplant patients with acute allograft rejection. Similarly, lower proportions of Vδ2 γδ T cells were observed in operationally liver transplant recipients, having not received immunosuppression for at least 12 months.^45^ However, these findings need to be interpreted with caution as an expansion of Vδ1 γδ T cells (thereby decreasing the proportion of Vδ2 γδ T cells) was observed following liver and kidney transplantation, regardless of immunosuppression treatment. It is possible that Vδ1 T clonotypes expand in the blood as a result of post‐transplant infections, such as CMV, as reported in healthy individuals^46^ and following transplantation.

While γδ T cells may contribute to the control of post‐transplant infection to enhance clinical outcomes, the co‐expression of CD16 may allow them to participate in antibody‐mediated rejection. One study found the expansion of CD16^+^ γδ T cells in kidney transplant patients with donor‐specific antibodies was associated with renal dysfunction.^47^ However, in patients without donor‐specific antibodies, such γδ T cells seem to be correlated with positive outcomes following transplantation, because of their ability to control CMV.

## Evidence for γδ T cells in favorable outcomes following transplantation

### γδ T cells in the control of post‐transplant CMV infection

γδ T cells have been implicated in the control of several pathogens, including tuberculosis, bacterial meningitis, human immunodeficiency virus and hepatitis C virus.^8^ However, CMV is the most common infectious complication following transplantation and γδ T cells are emerging as a significant player in the immunity to CMV. Following murine CMV (MCMV) infection, γδ T cells prevented an increase in viral load in all organs and were as effective as αβ T cells at controlling viral load in the lungs.^48^ The same authors also showed that transfer of MCMV‐induced γδ T cells into mice lacking innate and adaptive lymphocytes rescued the animals from MCMV‐induced death, indicating that γδ T cells were important in the response to MCMV.^48^ Another study confirmed that γδ T cells can effectively control MCMV in the absence of CD4^+^ T cells, CD8^+^ T cells and B cells.^49^

Following kidney transplantation in humans, reactivation of CMV drives a persistent expansion of γδ T cells expressing predominantly Vδ1 and Vδ3 TCR, collectively referred to as Vδ2‐negative γδ T cells^50^ (Figure 1). Their expansion parallels that of CMV‐specific CD8^+^ T cells,^51^ often resulting in an increase from 1% of circulating T cells to more than 10% of the total lymphocyte count.^52^ The expanded CMV‐specific Vδ2‐negative γδ T cells persisted for more than 1 year in kidney transplant recipients^53^ and their presence correlated with the resolution of viraemia, whereas their absence was associated with recurrent CMV disease.^54^ Similar to CMV‐specific CD8^+^ T cells, CMV‐specific γδ T cells possess an effector memory phenotype, in contrast to CMV‐negative patients, where they exhibited a naïve phenotype.^55^ Both effector memory Vδ2‐negative γδ T cells and CMV‐specific CD8^+^ αβ T cells of CMV‐infected renal transplant patients produced high levels of perforin, granzyme B, and expressed the activating NK cell receptor NKG2D. They appeared to be fully differentiated effector cells with a lower surface expression of CD28 compared to naïve T cells.^52^ Not only do effector memory Vδ2‐negative γδ T cells have the same differentiated effector phenotype as CD8^+^ αβ T cells, but they expand more rapidly in patients with CMV reactivation as opposed to primary CMV infection, which suggests that they may have an adaptive memory function.^55^ The persistent expansion of Vδ2‐negative γδ T cells following CMV infection, coupled with their differentiation into an effector/memory phenotype with expression of cytotoxic agents, implies that γδ T cells respond to CMV in an adaptive manner similar to cytotoxic CD8^+^ T cells. Like CD8^+^ T cells, recognition of CMV‐infected targets by Vδ2 negative γδ T cells is TCR‐dependent, although this occurs independent of MHC.^56^ The nature of the ligand(s) for Vδ2‐negative γδ T cells remains unknown but may include EPCR.^21^ However, EPCR expression is not upregulated by CMV infection and recognition of target cells by EPCR‐reactive clones requires costimulatory ligands.^21^

Unlike CD8^+^ αβ T cells, Vδ2‐negative γδ T cells may have the capacity to contribute to CMV immune control via antibody‐dependent cell‐mediated cytotoxicity (ADCC). CD16 is expressed by the majority of CMV‐induced γδ T cells, whereas it is expressed only by a small amount of Vδ2‐negative γδ T cells in renal transplant patients without CMV, suggesting that CD16 on Vδ2‐negative γδ T cells is upregulated in the response to CMV.^19^ However, the presence of CD16^+^ Vδ2‐negative γδ T cells may be problematic in transplant recipients with donor‐specific antibodies because of their ability to lyse antibody‐coated target cells.^51^

The expansion of CMV‐specific Vδ2‐negative γδ T cells was first observed in kidney transplant recipients but has subsequently been shown to occur in heart and lung transplant recipients^57^ and following HSCT.^58^ Longitudinal monitoring of γδ TCR repertoires in HSCT patients using next‐generation sequencing revealed that the CMV‐induced Vδ2‐negative γδ T cells were clonal in nature.^59^ Reactivation of CMV following HSCT induced significant changes in both the TRG (TCRγ) and TRD (TCRδ) repertoires. There were no public or shared sequences specific to CMV, as individual patients had distinct clonal γδ TCR responses to CMV, although there was some homology.^59^ Another study also showed that the TRD repertoire had reduced diversity in patients with CMV, further demonstrating the remarkable impact CMV can exert on γδ T cells.^60^

### γδ T cells in the control of post‐transplant malignancies

Interestingly, in addition to their antiviral function, CMV‐induced Vδ2‐negative γδ T cells have been associated with reduced occurrence of skin and solid cancers in kidney transplant patients.^61^ Patients who had not experienced CMV infection either prior to or following transplantation, and therefore lacked CMV‐induced γδ T cells, experienced a higher rate of malignancies. The expansion of CMV‐specific Vδ2‐negative γδ T cells was associated with reduced cancer occurrence, and these CMV‐specific Vδ2‐negative γδ T cells were shown to be able to kill tumor cells as efficiently as CMV‐infected cells *in vitro*.^51^ Akin to recognition of CMV‐infected cells, the killing of tumor targets by Vδ2‐negative γδ T cells was dependent on TCR engagement.^56^ This implies that CMV infection and transformation causes the upregulation of a common antigen that is recognised by the TCR of Vδ2‐negative γδ T cells. This phenomenon is not restricted to kidney transplant patients, as CMV‐associated Vδ2‐negative γδ T cells show anti‐leukaemic effects following HSCT.^62^, ^63^ However, the anti‐leukaemic effector functions of Vδ1‐positive γδ T cells were only partially dependent on TCR and strongly dependent on the expression of B7‐H6, a ligand for the NK cell receptor NKp30.^64^

Vδ2‐positive γδ T cells, in particular the Vγ9Vδ2 subset, have also been found to exert anti‐tumor effects. Vγ9Vδ2 cells isolated from the blood of patients following HSCT can be expanded *in vitro* and efficiently lyse lymphoid and myeloid targets.^63^ This subset is selectively expanded *in vitro* by phosphoantigen stimulation following exposure of cells to zoledronic acid.^18^ The *in vivo* activity of the Vγ9Vδ2 subset can be further boosted by direct infusion of zoledronic acid to the patient. These features have seen clinical trials of Vγ9Vδ2 γδ T cells in cell therapy for the treatment of solid tumors and haematological malignancies.^18^

Additionally, CD16^+^ Vγ9Vδ2 γδ T cells have been shown to lyse lymphoma, chronic lymphocytic leukaemia and breast cancer cells coated with antibodies via ADCC.^65^ Moreover, γδ T cells were shown to have a beneficial role against refractory leukaemia by specifically targeting the recipient's cancer cells without GvHD.^66^ Taken together, the data suggest that γδ T cells are efficient in controlling post‐transplant malignancies by multiple mechanisms including direct recognition of tumor antigens, ADCC and through the recognition of stress‐associated antigens.

### Suppression of post‐transplant immune responses by γδ T cells

γδ T cells may also contribute to favorable outcomes through suppression of immune responses. Lower proportions of CD8^+^ regulatory γδ T cells were found in the blood of renal transplant recipients with acute or chronic rejection.^67^ Similarly, higher numbers of CD8^+^ regulatory γδ T cells in renal allografts were associated with prolonged survival in a rat model of renal transplantation.^68^ The proposed mechanism is through the production of IL‐4 and IL‐10 from CD8^+^ regulatory γδ T cells, which acts to effectively dampen Th1 responses. Supporting this notion, improved graft survival was associated with expansions of γδ T cells and the increased production of IL‐4 and IL‐10 in an animal model of skin transplantation.^69^ IL‐4 in turn has a profound effect on the γδ T cell population and favors the survival of IL‐10‐producing Vδ1 cells.^70^ Improved survival in this model was lost following the administration of an antibody to γδ TCR. Interestingly, the production of IL‐10 from Vδ1 γδ T cells has been hypothesised to induce operational tolerance following paediatric liver transplantation.^71^ Likewise, higher proportions of regulatory Vδ1 γδ T cells that co‐expressed CD4 and CD25 were found in the blood of tolerant adult liver transplant recipients.^45^ Therefore, both animal models and human studies indicate regulatory γδ T cells can positively contribute to engraftment following transplantation, possibly by the production of IL‐4 and/or IL‐10.

An increase in regulatory γδ T cells also reportedly reduces the occurrence of GvHD following HSCT. Novel subsets of regulatory γδ T cell that express Foxp3 were associated with lower GvHD in HSCT patients.^72^ Interestingly, the Foxp3‐positive subsets utilised both Vδ1 and Vδ2 TCR segments, and a follow‐up study narrowed the effective subset to be CD27^+^Vδ1^+^.^73^ However, in direct contrast, grafts containing higher proportion of CD8^+^ γδ T cells were associated with increased incidence of GvHD.^74^ Therefore, as reported in the above section, the role of γδ T cells in the prevention or promotion of GvHD following HSCT is far from clear.

## Conclusions and future directions

γδ T cells represent an under‐researched population of immune cells with the propensity to significantly contribute to adverse and positive outcomes following transplantation, via both innate and adaptive pathways (Figure 1). However, as the underlying cause of transplantation and the infectious insults following transplantation vary widely between recipients, the role of γδ T cells needs to be carefully evaluated in the specific context.

Adverse functions of γδ T cells appear to be largely linked to the production of IL‐17. On the one hand, CD16^+^, CMV‐specific cells may exert ADCC on transplanted cells coated in donor‐specific antigens, thereby contributing to antibody‐mediated rejection. On the other hand, these same CMV‐specific γδ T cells effectively control viral replication and post‐transplant malignancies. Furthermore, other γδ T cell subsets can efficiently suppress adaptive immune responses and aid in immune tolerance following transplantation. The role of γδ T cells in preventing or promoting GvHD following HSCT is highly controversial and may be dependent on different subsets exerting opposite effects.

Although the role of particular subsets of γδ T cells is dependent on the individual context, it is clear these cells are an active and dynamic component of the transplant environment. An identification of the ligands for γδ T cells will significantly aid in harnessing their therapeutic potential following transplantation. Indeed, more research is required to unveil specific subsets of γδ T cells with a view to develop novel therapies that can meaningfully contribute to positive outcomes following transplantation.

## Conflict of interest

The authors declare no conflict of interest.
